# Ein Scharnier zwischen Eugenik und Recht – der „medizinisch-juristische“ Kommentar im „Dritten Reich“

**DOI:** 10.1007/s00048-021-00307-6

**Published:** 2021-08-24

**Authors:** Vivian Yurdakul

**Affiliations:** 1 Institut für Geschichtswissenschaften, Humboldt-Universität zu Berlin, Unter den Linden 6, 10099 Berlin, Deutschland; 2 Interdisziplinäres Zentrum für Wissenschafts- und Technikforschung, Bergische Universität Wuppertal, Gaußstraße 20, 42119 Wuppertal, Deutschland

**Keywords:** Gesetzeskommentar, „Drittes Reich“, „Rassenrecht“, Sachverständige, Ressourcenmobilisierung, Juristic Commentaries, Third Reich, Racial Law, Experts, “Mobilization of resources”

## Abstract

Ab 1934 traten vermehrt Wissenschaftler unterschiedlicher Disziplinen, insbesondere Mediziner, als Autoren einer vormals rein rechtswissenschaftlichen Fachtextgattung auf, nämlich des juristischen Kommentars. Der folgende Text geht am Beispiel des maßgeblichen, von zwei Ärzten und einem Juristen verfassten Rechtskommentars zum Blutschutz- und Ehegesundheitsgesetz von 1935 der Frage nach, wie sich dadurch die Gesetzeserläuterung veränderte. Hintergrund ist eine ab 1934 in den juristischen Fachzeitschriften geführte Debatte um eine Reform des Genres Gesetzeskommentar, das in seiner herkömmlichen Anlage als inkompatibel mit dem nationalsozialistischen Rechtsdenken galt.

Der Aufsatz versucht diesen Prozess in Ausweitung des Theorieansatzes Mitchell G. Ashs als „Verflechtung“ des Rechts mit anderen Wissenschaften zu begreifen, die zu beiderseitigem „Vorteil“ war: Auf der einen Seite konnte sich die Jurisprudenz auf vorgeblich „wissenschaftlich-objektive“ Befunde berufen, die, anders als juristische Abwägungen – so die Suggestion –, als „nicht hinterfragbar“ angesehen wurden. Andererseits verlieh die unmittelbare Rechtsrelevanz bestimmter Befunde aus den betreffenden Wissenschaften diesen Disziplinen insgesamt einen höheren gesellschaftlichen Stellenwert, der wiederum die Erschließung neuer Forschungsfelder ermöglichte.

Geprüft wird außerdem ein möglicher Zusammenhang mit einer partiellen Veränderung der Gerichtslandschaft: Mit der verstärkten Gesetzeskommentierung durch Nicht-Juristen erweiterte sich schließlich auch der Adressatenkreis der betreffenden Schriften. Auf Grundlage vieler ab 1933 geschaffener Gesetze wurden neue Gerichte ins Leben gerufen, an denen sogenannte sachkundige Laienrichter mitentschieden – „Fachleute“ aus den Disziplinen, auf die sich die Gesetzgebung nun berief.

## Abstract (en)

Before 1933 commentaries on laws were exclusively juristic texts, written and read only by legal professionals. Beginning in 1934, scholars from different disciplines, especially medical scientists, began writing juristic commentaries. The essay examines the reasons for this development and explores how it changed the genre, using the example of the most important commentary on the *Blutschutz-* and *Ehegesundheitsgesetz*, which resulted from the collaboration of two medical professionals and a legal professional. The article argues that the recruitment of non-juristic authors and the corresponding methodological changes were caused by a debate on a possible “reform” of juristic commentaries which took place in law journals. From the perspective of the National Socialist regime, the genre in its traditional form was not compatible with the Third Reich’s philosophy of law.

In this new genre, commentaries not only referred to legal problems, but also dealt with issues from other disciplines, such as medical questions. The essay modifies Mitchell G. Ash’s theoretical approach regarding the “entanglement” of jurisprudence and other disciplines. Law and other sciences became, according to Ash, “resources for one another”: Legal scholars could claim that legal decisions were based on scientific facts and therefore “unquestionable.” The affected disciplines also gained greater importance as a result of this process in which their findings becoming legally relevant. This transformation also enabled them to acquire resources for new research projects.

Moreover, the essay examines the connection between this development and the change of audience within German courts. Not only did the authors of the commentaries change, but also the recipients: Many laws passed after 1933 introduced new courts using lay judges, professionals with a non-law background. These “experts” came from multiple fields connecting them to the new law, such as medical professionals.

Die Wissenschaftsgeschichte hat in den letzten Jahren betont, dass der Machtantritt der Nationalsozialisten 1933 für natur- und technikwissenschaftliche Disziplinen eine weniger markante Zäsur darstellte, als lange angenommen (Hachtmann 2018b: 19; Hachtmann 2021). Anders verhielt es sich im Fall der dem jeweiligen politischen System traditionell nahestehenden „ideologienahen“ Wissenschaften. Insbesondere die Jurisprudenz stand nach der Machtübernahme der NSDAP zur Disposition. Ein Vierteljahr nach der Vereidigung Hitlers als Reichskanzler wurde Hans Frank zum „Reichskommissar für die Gleichschaltung der Justiz und für die Erneuerung der Rechtsordnung“ ernannt. Dies kann als eines unter vielen Indizien dafür gesehen werden, dass für das Rechtswesen von einer „Kontinuität nach 1933“ nicht auszugehen ist.

Angesichts dieser Identitätskrise war die Rechtswissenschaft gravierenden Transformationsprozessen unterworfen. Allerdings eröffneten die Veränderungen – die im Übrigen von einem Gros der Juristen befürwortet wurden (Lepsius 1994: 1–4) – auch neue Einflussmöglichkeiten; nicht nur der Rechtswissenschaft selbst, sondern ebenso anderen Disziplinen. Zugleich wurden diese Wissenschaftsfelder somit enger miteinander verflochten. Besonders das Verhältnis von Jurisprudenz einerseits sowie Medizin und Eugenik andererseits intensivierte sich: Die NS-Diktatur verstand sich als „Bio-Macht“ (Eckart 2012: 14) mit der Mission, den „Volkskörper“ von vermeintlich schädlichen Erbanlagen zu „reinigen“. Im eigens dafür geschaffenen „Rassenrecht“ zeichneten sich die Entwicklungen, die im Folgenden betrachtet werden, besonders deutlich ab. Zwar handelte es sich formal lediglich um eines von vielen ab 1933 neu geschaffenen Rechtsgebieten – daneben wären etwa „nationales Arbeitsrecht“, Erbhofrecht, Naturschutzrecht, Jagdrecht und Energiewirtschaftsrecht zu nennen. De facto kam dem „Rassenrecht“ in einem „Rassenstaat“ (Eckart 2012: 15) jedoch eine herausgehobene Position zu. Rechtswissenschaftler und Ärzte sahen ihren Schulterschluss zur Umsetzung der faschistischen Gesundheitspolitik daher bereits in der Anlage des „neuen Staates“ präfiguriert. In dieser engen Kooperation kam zum Ausdruck, dass die Ehe- und Sexualpolitik im Nationalsozialismus, wie Gabriele Czarnowski zugespitzt hat, „nicht rückwärtsgewandt, reaktionär oder konservativ war, sondern ganz im Gegenteil höchst modern“ (Czarnowski 1991: 15) – „modern“ in einem negativen Sinne, versteht sich, indem sie technokratische und biologistische Vorstellungen vereinte.

Überraschend wurde ausgerechnet der Gesetzeskommentar, eine der wichtigsten Fachtextgattungen der Jurisprudenz, ab 1934 zu einem „Scharnier“, über das Disziplinen – insbesondere eben Medizin und Eugenik – Einfluss auf das Rechtswesen gewannen, die zuvor kaum Berührungspunkte mit ihm gehabt hatten. Im Umkehrschluss wuchs über das Medium des Kommentars zugleich der Einfluss der Jurisprudenz auf die betreffenden Wissenschaften (Yurdakul 2017). Erstaunlich war dies, weil sich die generelle Kritik der Nationalsozialisten an der überkommenen Rechtswissenschaft zunächst besonders am Genre der juristischen Erläuterung entladen hatte. Nach der Machtübergabe an Hitler fand in juristischen Medien eine intensive Debatte um eine Reform dieser Textsorte statt (Seydel 1936; Kästle 2013; Kästle-Lamparter 2016: 76–84).

Kommentare sind Erläuterungen, in denen die Verfasser Gesetze auslegen, indem sie deren Wortlaut deuten, Lesarten abwägen und Urteile heranziehen, die auf ihrer Grundlage ergangen sind. Autoren wie Rezipienten sind ergo in erster Linie Juristen. Relevant sind Kommentare besonders für die Rechtspraxis, da sich Rechtsparteien auf sie berufen und Gerichte sie zur Entscheidungsfindung heranziehen.

Als „zentrale[s] Medium juristischer Kommunikation“ (Kästle-Lamparter 2016: 70) erlebte der Kommentar in der Weimarer Republik eine Blüte (Kästle-Lamparter 2016: 73f.). Dies war darauf zurückzuführen, dass er besonders geeignet schien, wesentliche Forderungen der zu dieser Zeit führenden positivistischen Rechtsschule zu erfüllen. Diese betrachtete Rechtssicherheit als oberstes Ziel des Rechtswesens; sie forderte deshalb eine strikte Bindung an kodifiziertes Recht und sprach sich für eine streng wortlautorientierte Gesetzesauslegung aus (Grunert 2008). Entsprechend galt der Kommentar, dessen wesentlicher Inhalt in einer ebensolchen Wortlautauslegung bestand, als positivistische Textgattung schlechthin.

Dass die Auslegungsschriften stark mit der in der Weimarer Demokratie dominierenden Rechtstheorie assoziiert waren, sorgte dafür, dass die Nationalsozialisten Anstoß an ihnen nahmen (Kästle 2013: 432). Die NS-Juristen lehnten den Positivismus und alles, was mit ihm in Verbindung stand, ab (Wittreck 2008: 3) und stellten ihm die Idee eines sogenannten „lebensgesetzlichen Rechts“ gegenüber. Dieses sollte auf vorgeblich objektiven wissenschaftlichen Befunden beruhen und damit – anders als Wortlautinterpretationen und rechtstheoretische Erwägungen – vermeintlich nicht hinterfragbar sein (Wittreck 2008: 43–45). Zudem sollte die Ausrichtung an angeblich objektiven Forschungserkenntnissen aus unterschiedlichsten Disziplinen eine Zwangsläufigkeit der entsprechenden Beschlüsse suggerieren. Die Entscheidung darüber, was als rechtlich richtig und falsch gelten konnte, sollte ersetzt werden durch eine apodiktische Feststellung dessen, was „wissenschaftlich erwiesenermaßen notwendig“ sei. Auf diese Weise sollte ein Rechtssystem entstehen, das den Ansprüchen der Diktatur entsprach, indem es wie diese keinen Widerspruch duldete.

Für den Kommentar bedeutete dies, dass an den Abwägungen widerstreitender Urteile und Lesarten von Gesetzen, die bis dato sein Hauptgegenstand gewesen waren, im „neuen Recht“ eigentlich kein Bedarf mehr war. Vereinzelt wurde daher ein radikales Verbot der Auslegungsschriften gefordert (Ludwig 1934: 29; Kästle 2013: 432). Solche ideologisch motivierten Bestrebungen kollidierten aber mit rechtspraktischen Erfordernissen: Kommentare waren ein so wichtiges Handwerkszeug des juristischen Alltags, dass ihre Abschaffung „unrealistisch“ schien (Kästle-Lamparter 2016: 78). Dass der Kommentar in seiner althergebrachten Form inkompatibel mit den nationalsozialistischen Rechtsvorstellungen, ein Rechtswesen ohne ihn jedoch kaum denkbar war, prädestinierte ihn ironischerweise wiederum, zu einem tragenden Medium der Neuausrichtung des Rechts im NS-Staat zu werden. In Fachzeitschriften wurde ab 1934 diskutiert, wie mit der Textgattung umzugehen sei. Im Zuge dieser Debatte, die sich bis in die zweite Hälfte der 1930er Jahre intensivierte (Hedemann 1935/36; Seydel 1936; Siebert 1936: 80),^1^ kristallisierte sich der Konsens heraus, das Kommentarwesen müsse einer Neuausrichtung unterzogen werden.^2^ Konkrete Vorstellungen, wie die avisierte Reform aussehen sollte, äußerten die Autoren der Diskussionsbeiträge allerdings nicht. So hielt der NS-Rechtswissenschaftler Wolfgang Siebert (1905–1959) nur fest: „Wie weit diese Aufgabenstellung eine Veränderung der bisher üblichen Formen für den Aufbau der Erläuterung mit sich bringen muß, soll hier nicht näher untersucht werden“ (Siebert 1936: 80).

Die historische Forschung hat sich mit dem daraus resultierenden Wandel des juristischen Kommentars bislang schwergetan.^3^ Neuere Studien räumen ein, dieser Themenkomplex „müsste näher untersucht werden“ (Kästle-Lamparter 2016: 84). Im folgenden wird der Frage nachgegangen, wie sich die Gesetzeserläuterung ab 1933/34 entwickelte und welche Rolle rechtswissenschaftsfremde Disziplinen dabei spielten. Die methodologische Ausgangsthese lautet, dass übliche rechtshistorische Herangehensweisen nicht greifen, will man die veränderte Methodik des juristischen Kommentierens im Nationalsozialismus fassen. Vielmehr legt eine Reihe an Befunden nahe, dass eher Modelle der Wissenschaftsgeschichtsschreibung geeignet sind, den Wandel der Auslegungspraxis einzuordnen, da sich der Kreis der Verfasser wie Adressaten juristischer Erläuterungen markant erweiterte.

Ab 1934 wirkten vermehrt Autoren ohne rechtswissenschaftliche Ausbildung an den eigentlich juristischen Schriften mit. Sie entstammten den Disziplinen, aus deren Erkenntnissen die NS-Gesetze vorgeblich abgeleitet wurden, und traten häufig als maßgebliche Herausgeber führender Kommentare auf, während die beteiligten Juristen auf den Titelblättern nunmehr an letzter Stelle genannt wurden. Dies lässt sich, wie angedeutet, besonders auf dem Gebiet des „Rassenrechts“ beobachten, auf dem vor allem der Medizinalbeamte Arthur Gütt (1891–1949) sich und weiteren Ärzten den Weg in die Autorenkollegien juristischer Erläuterungen ebnete (z. B. Gütt et al. 1934; Gütt & Moebius 1935). Aber etwa auch das Gesetz zur Ordnung der nationalen Arbeit vom 20. Januar 1934 (Reichsministerium des Innern 1934a) wurde mit Wolfgang Pohl von einem Arbeitswissenschaftler und Nationalökonomen mitkommentiert (Mansfeld et al. 1934).

Die Rechtsgeschichte begnügte sich bisher weitgehend damit, die unterschiedliche Kommentierung systemübergreifend geltender Gesetze durch Juristen in der Weimarer Republik, dem „Dritten Reich“ und der Nachkriegszeit zu vergleichen (Kästle 2013: 433–436; Kästle-Lamparter 2016: 81–84). So erklären sich Befunde wie der, „dass die juristischen Kommentare während der NS-Zeit trotz ihrer ideologischen Neuorientierung im Wesentlichen das blieben, was sie waren“ (Kästle-Lamparter 2016: 84). Will man aber zum Kern der NS-„Kommentarreform“ vordringen, gilt es, Erläuterungen genuin nationalsozialistischer Gesetze zu betrachten, namentlich solche, an denen neben Juristen Wissenschaftler anderer Fachrichtungen beteiligt waren. Dies legen die zeitgenössischen Artikel zum Thema nahe, die in juristischen Zeitschriften erschienen. So nannte etwa der maßgeblich in die Debatte um neue Funktionen des Kommentars involvierte Rechtsanwalt Helmut Seydel als „gute[s] Beispiel[…]“ (Seydel 1936: 192) dafür, wie der (nationalsozialistische) Rechtskommentar künftig aussehen könnte, eine von den Medizinern Arthur Gütt und Ernst Rüdin (1874–1952) sowie dem Juristen Falk Ruttke (1894–1955) verfasste Erläuterung des Gesetzes zur Verhütung erbkranken Nachwuchses (GzVeN) vom 14. Juli 1933 (Reichsministerium des Innern 1933, im Folgenden paragraphengenau zitiert unter der Sigle GzVeN).

Die vermehrte Gesetzeserläuterung durch Nicht-Juristen ist vor dem Hintergrund einer sich verändernden Rechts- und Gerichtslandschaft im „Dritten Reich“ zu verstehen: Analog zum Kreis der Autoren erweiterte sich der Kreis der Rezipienten. Der NS-Staat band in die Gesetzesanwendung verstärkt als „sachkundig“ bezeichnete juristische Laien als Entscheidungsträger ein. Insbesondere verhalf er dem Stand des „sachverständigen Laienrichters“ zur Hochkonjunktur (Yurdakul 2020). Diese besondere Form des richterlichen Beisitzers, der sich statt durch eine juristische Ausbildung durch seine Fachkompetenz auf dem zu verhandelnden Sachgebiet qualifiziert, hatte es zuvor nur vereinzelt vor allem an Handels- und Arbeitsgerichten gegeben. Ab 1934 wurde eine ganze Reihe neuartiger Gerichte aus der Taufe gehoben, auf deren Richterbänken vermeintliche Experten ohne juristisches Studium Platz nahmen, um auf Grundlage bestimmter neu geschaffener Gesetze Entscheidungen zu treffen.^4^ So beschlossen in den durch das GzVeN begründeten Erbgesundheitsgerichten zwei Mediziner gemeinsam mit einem Juristen Zwangssterilisationen (§ 6 Abs. 1 GzVeN) und Eheverbote (Reichsministerium des Innern 1935c: 1420, hier § 11 Erste DurchfVO z. Ehegesundheitsgesetz). Bisher existieren zu den Gerichten, die unter Beteiligung sachkundiger Laien entschieden – das heißt unter Mitwirkung ehrenamtlicher Richter, „die über eine gewisse Sachkunde auf dem besonderen Arbeitsgebiet des Gerichts verfügen“ (Schafheutle 1937: 132) – keine übergreifenden Untersuchungen. Sie wurden, wenn überhaupt, lediglich vereinzelt und meist auf lokaler Ebene in den Blick genommen.^5^

## Die Verschränkung von Wissenschaft und Recht im Kommentar „neuen Typs“

Der vorliegende Aufsatz knüpft an eine jüngere Untersuchung an, die am Beispiel der erwähnten Erläuterung zum GzVeN der Mediziner Gütt und Rüdin sowie des Juristen Ruttke zeigt, wie die in dieser Konstellation angelegte Verflechtung der Jurisprudenz mit anderen Wissenschaften zu einer stärkeren Einbindung von „Experten“ aus fremden Disziplinen auch in die juristische Kommentierung führte und welche Folgen daraus erwuchsen (Yurdakul 2017): Der Kommentar wurde zu dem Ort, an dem sich die besagte Verflechtung praktisch vollzog. Analog zu der von Mitchell G. Ash herausgearbeiteten „Verwissenschaftlichung der Politik“ (Ash 2002: 38) im Nationalsozialismus, zu der eine „Politisierung der Wissenschaften“ (Ash 2002: 38) parallel lief, kam es, so die These, durch das Medium des Kommentars zu einer „Einflussnahme der wissenschaftlichen auf die rechtliche Sphäre“, die mit einer „Einflussnahme der rechtlichen auf die wissenschaftliche Sphäre“ einherging (Yurdakul 2017: 103–109). Fachwissenschaftliche – im Fall des GzVeN medizinische – und juristische Begrifflichkeiten, Theorien und Methoden des Erkenntnisgewinns verschmolzen im Kommentar: Im Rahmen der Auslegung originär juristischer Termini und der Beantwortung juristischer Fragen wurden Theorien sowie Methoden aus anderen Wissenschaften als der Jurisprudenz herangezogen und fachwissenschaftliche Begriffe zu (häufig unbestimmten) Rechtsbegriffen umgedeutet. Auf der anderen Seite wurden rechtswissenschaftliche Denk- und Argumentationsfiguren (wie rechtliche Gleichstellungen und Analogien) sowie Methoden angewandt, um genuin fachwissenschaftliche Fragen zu beantworten (Yurdakul 2017: 106). Das vorgeblich nur juristische Instrumentarium des Gesetzeskommentars entfaltete somit auch eine Wirkung auf die betreffenden Wissenschaften. Beide Formen der Einflussnahme sind nicht zu trennen. Es handelte sich um einen Prozess, in dem juristische und fachspezifische Begrifflichkeiten sich „amalgamierten und wechselseitig radikalisierten“ (Vormbaum 2017: XIII). Dies geschah – gleichfalls analog zum Ash’schen Modell, demzufolge Wissenschaften und Politik zu „Ressourcen füreinander“ wurden (Ash 2002: 40) – zum beiderseitigen „Vorteil“ von Rechts- und „Fach-“Wissenschaft; im Falle des GzVeN etwa der Medizin: Einerseits verlieh die Rechtsrelevanz bestimmter wissenschaftlicher Befunde der Disziplin, aus der sie hervorgegangen waren, einen höheren gesellschaftlichen Stellenwert. Dies ermöglichte die Erschließung neuer Forschungsfelder. Andererseits konnten sich Jurisprudenz und Justiz im Rahmen der Entscheidungsfindung auf angeblich wissenschaftlich-objektive Erkenntnisse berufen. Damit begegnete der Kommentar „neuen Typs“ einem wesentlichen Kritikpunkt der Nationalsozialisten am vor 1933 dominanten Positivismus. Die Nazis störten sich vor allem an dessen Begriffsbildung, die vorsah, ein Rechtsbegriff sollte „entsprechend dem Zweck der jeweiligen Rechtsnorm und unter Umständen auch abweichend von seiner fachspezifischen Bedeutung bestimmt werden“ (Schröder 2016: 45). Hingegen suchte die vorgeblich wissenschaftlich-objektive „lebensgesetzliche“ Rechtslehre „nach dem *einen* [Hervorhebung im Original] richtigen, wesensentsprechenden Begriff“ (Schröder 2016: 45), der nicht mehr zu hinterfragen war. Indem er wissenschaftliche Fachbegriffe zu Rechtsbegriffen umdeutete, die in ihrer Bedeutung dezidiert mit dem (dann häufig seinerseits veränderten) fachwissenschaftlichen Sinngehalt übereinstimmten, trug der Kommentar „neuen Typs“ dazu bei, die mit dem NS-Rechtsdenken zunächst inkompatible Jurisprudenz anschlussfähig an den „Neuen Staat“ zu machen.

Gewährleistet war die Kongruenz von Fach- und Rechtsbegriff, da die Kommentare „neuen Typs“ für Wissenschaftler wie Juristen faktisch verbindlich waren. Sie waren im Gegensatz zu konventionellen Erläuterungsschriften – die nicht ausstarben, sondern auf allen Rechtsgebieten parallel weiterexistierten – von einem Nimbus der Amtlichkeit umgeben. Das war ebenfalls auf die Mitwirkung der Nicht-Juristen an der Gesetzesanwendung zurückzuführen: Die juristischen Laien wurden häufig von offiziellen Stellen zur Anschaffung der betreffenden Schriften verpflichtet (Gaupp 1934: 780). Bei der Anwendung der Vorschriften spielten die „sachverständigen Richter“ die entscheidende Rolle, weshalb die Juristen in der Frage, welche Kommentare in der Rechtspraxis gängig werden sollten, nicht über sie hinweggehen konnten. So gelang es dem Regime, die bis dato rechtswissenschaftsinternen Aushandlungsprozesse darüber, welche Auslegung sich durchsetzte, auszuhebeln (Yurdakul 2017: 116f.). Es wäre jedoch falsch, daraus zu schlussfolgern, die Juristen hätten die mit der NS-Ideologie höchst konformen Schriften abgelehnt – im Gegenteil: Die rechtswissenschaftliche Fachliteratur reagierte euphorisch auf sie (Seydel 1934: 1037f.; Schmidt-Klevenow 1936b: 511f.).

Im Folgenden wird geprüft, ob die am Beispiel der GzVeN-Erläuterung entwickelte These einer Verschränkung von Wissenschaften und Recht sowie einer wechselseitigen Ressourcenmobilisierung durch eine neue Form der Rechtskommentierung allgemeinere Gültigkeit beanspruchen kann. Auf der methodologischen Ebene soll damit ein erster Beitrag zur Beantwortung der Frage geleistet werden, inwieweit sich Ashs Konzept einer Verflechtung von Wissenschaften und Politik ausweiten lässt (Hachtmann 2018a), um mit ihm das Verhältnis der Wissenschaften zu anderen gesellschaftlichen Subsystemen wie auch die Beziehungen der Forschungsdisziplinen untereinander zu analysieren. Denn da Recht gleichermaßen Profession wie Wissenschaft ist, erfolgte dessen Verflechtung mit der Medizin auf zwei Ebenen: Einerseits verband sich das Rechtswesen als soziales Teilsystem mit dem sozialen Teilsystem Wissenschaft. Grundlage dafür war andererseits eine Verschmelzung der Jurisprudenz als Disziplin mit der Medizinwissenschaft, also eine Verschränkung zweier Wissenschaften. Zugleich ist die von Ash betonte politische Dimension als Metaebene stets mitzubedenken, da sich all diese Verflechtungen mit Blick auf die politischen Ziele des NS-Regimes vollzogen.

Die eingangs skizzierte Debatte um den Kommentar im Allgemeinen lässt vermuten, dass der Gütt/Rüdin/Ruttke kein Einzelfall war, sondern sich die dort beobachtete unkonventionelle Auslegungstechnik auch in anderen Erläuterungen findet. Zugleich soll gefragt werden, wo die Grenzen dieses Phänomens zu verorten sind. Verschränkten sich Wissenschaften und Recht nur in der Kommentierung jener Gesetze, deren Einhaltung sachkundige Laien mitkontrollierten? Dies legen Äußerungen wie die des NS-Juristen Justus Hedemann nahe. Er knüpfte seine Hoffnungen auf eine Kommentarreform kaum zufällig an die „schönen neue[n] Rechtsgebiete“, an „Erbhofrecht, nationales Arbeitsrecht usw.“ und damit an Rechtsfelder, deren Gemeinsamkeit in der Beteiligung sachkundiger Laien – Landwirten sowie Unternehmern und sogenannten betrieblichen Vertrauensleuten – bestand (Hedemann 1935/36: 142).

Hier kann wie gesagt nicht die Kommentierung auf all diesen Gebieten thematisiert werden. Welche Änderungen der Erläuterungsmethodik die Diskussion um die Textgattung Kommentar nach sich zog, wird daher exemplarisch anhand des maßgeblichen Kommentars zum Blutschutz- und Ehegesundheitsgesetz analysiert. Dieser wurde von Arthur Gütt, dem Mediziner Herbert Linden (1899–1945) sowie dem Juristen Franz Maßfeller (1902–1966) publiziert (Gütt et al. 1936, im Folgenden zit. unter der Sigle GLM). Die „Doppelerläuterung“ bietet sich aus zwei Gründen als Gegenstand an: Erstens erschien sie im gleichen Verlag wie der Gütt/Rüdin/Ruttke und wurde von vielen Rezensenten als dessen Ergänzung wahrgenommen (Gaupp 1937: 209), ebenso wie das Ehegesundheitsgesetz (Reichsministerium des Innern 1935d, im Folgenden paragraphengenau zitiert unter der Sigle EGG) selbst als Fortsetzung des GzVeN mit anderen Mitteln bezeichnet werden könnte. So besteht ein inhaltlicher Bezug zu der Untersuchung, an die der vorliegende Text anknüpft. Zum anderen waren für „Ehegesundheitsangelegenheiten“ ebenfalls die mit Ärzten besetzten Erbgesundheitsgerichte zuständig, für „Blutschutzangelegenheiten“ aber Standesämter und Amtsgerichte – also Instanzen, die ohne sachkundige Laien auskamen. Daher lässt sich anhand eines Vergleichs der Erläuterungen beider Gesetze der Frage nachgehen, ob eine neue Kommentierungsmethodik nur auf Rechtsgebieten feststellbar ist, auf denen sachkundige Laien zum Einsatz kamen.

## Das Blutschutz-, das Ehegesundheitsgesetz und der Kommentar von Gütt, Linden und Maßfeller

Das Blutschutz- und das Ehegesundheitsgesetz zählten zu den 1935 verabschiedeten „Rassegesetzen“. Das als eines der Nürnberger Gesetze erlassene Blutschutzgesetz (Reichsministerium des Innern 1935e, im Folgenden paragraphengenau zitiert als „Blutschutzgesetz“) verbot in erster Linie Ehen (§ 1) zwischen Juden im Sinne des Gesetzes und sogenannten „Staatsangehörigen deutschen oder artverwandten Blutes“. Daneben enthielt es weitere diskriminierende Restriktionen.

Das rund einen Monat später verabschiedete EGG untersagte Eheschließungen, wenn einer der Partner

- „an einer mit Ansteckungsgefahr verbundenen Krankheit [litt], die eine erhebliche Schädigung der Gesundheit des anderen Teiles oder der Nachkommen befürchten“ ließ (§ 1 Abs. 1 Buchst. a),
- entmündigt war oder unter vorläufiger Vormundschaft stand (§ 1 Abs. 1 Buchst. b),
- „ohne entmündigt zu sein an einer geistigen Störung [litt], die die Ehe für die Volksgemeinschaft unerwünscht erscheinen“ ließ (§ 1 Abs. 1 Buchst. c),
- unter einer „Erbkrankheit“ im Sinne des GzVeN litt (§ 1 Abs. 1 Buchst. d).

Letztgenanntes Verbot fand keine Anwendung, wenn beide Partner unfruchtbar waren (§ 1 Abs. 2).

Zur Durchsetzung der Eheverbote sollten reichsweit alle Verlobten vor der Eheschließung ein von den Gesundheitsämtern auszustellendes Ehetauglichkeitszeugnis einholen. Dieses musste bescheinigen, „daß ein Ehehindernis nach § 1 nicht vorliegt“ (§ 2). § 2, der das Tauglichkeitszeugnis zur Pflicht erklärte, trat allerdings nicht unmittelbar in Kraft. Gemäß § 8 Abs. 2 sollte der Reichsinnenminister den Zeitpunkt des Inkrafttretens frei bestimmen. Bis dahin war ein Ehetauglichkeitszeugnis „nur in Zweifelsfällen“ (§ 8 Abs. 2 EGG) vorzulegen. Tatsächlich kam es nie zu einer vollumfänglichen Inkraftsetzung von § 2.

Die mit Abstand auflagenstärkste Erläuterung der Gesetze, die den größten Einfluss auf die „Ehegesundheitsverfahren“ hatte (Klemm 1936: 1669; Meinhof 1936: 3446; Schmidt-Klevenow 1936a: 511), stellte der Kommentar von Gütt, Linden und Maßfeller dar. Sämtliche im Deutschen Reich tätigen Ärzte mussten ihn besitzen (O. A. 1940: 39); Mediziner nahmen ihn als „amtlich[…]“ (Schnell 1938: 134) wahr. Dass er bislang kaum in den Fokus der historischen Forschung gerückt ist, liegt daran, dass lange der von Hans Globke, dem späteren Staatssekretär Konrad Adenauers, mitverfasste Kommentar zu den beiden Gesetzen (sowie dem Reichsbürgergesetz) (Stuckart & Globke 1936) als der wichtigste wahrgenommen wurde (Rethmeier 1995: 135). Dessen tatsächliche Relevanz im „Dritten Reich“ wurde erst durch die jüngere Forschung relativiert (Wesel & Beck 2013: 161f.; Kästle-Lamparter 2016: 80):

Viele ältere Studien schließen aus der bedeutsamen Auseinandersetzung mit Globkes Erläuterung im Rahmen der Aufarbeitung personeller Kontinuitäten nach 1945 fälschlich, die Schrift hätte auch in der NS-Zeit den größten Einfluss auf die Rechtsprechung gehabt (Wesel & Beck 2013: 164). Begünstigt wurde dieser Fehlschluss dadurch, dass, wie im Folgenden zu zeigen ist, die eigentlich dominante Auslegung von Gütt, Linden und Maßfeller mit rechtshistorischen Ansätzen kaum greifbar ist. Ihr „Erfolg“ speiste sich gerade daraus, in weiten Teilen eben nicht juristisch zu argumentieren. Zugespitzt ließe sich sagen, dass der Gütt/Linden/Maßfeller-Kommentar von der Historiografie bisher aus dem gleichen Grund weitgehend ignoriert wurde, aus dem er damals so wirkmächtig war.

Bereits das Titelblatt des Buches liefert einen Hinweis darauf, dass sich die Autoren des hybriden Charakters ihres an einer Schnittstelle von eugenisch-medizinischer und juristischer Fachliteratur angesiedelten Werks bewusst waren: Dort heißt es, Blutschutz- und Ehegesundheitsgesetz würden „medizinisch und juristisch erläutert“ (GLM: III). Auch die spärliche Forschungsliteratur, die den Kommentar als Quelle heranzieht, bestätigt dieses „Selbstverständnis“ der Schrift – freilich ohne den Fokus auf die Bedeutung ihrer Identifikation als „medizinisch-juristische[n]“ (Czarnowski 1991: 69) Fachtext zu legen.

Ebenso hoben zeitgenössische Rezensionen die neue Herangehensweise hervor, die bei ihnen auf positive Resonanz stieß, da durch sie „weder dem Juristen noch dem Mediziner nur das ihm eigene Gebiet erschlossen wird, sondern beider Arbeiten sich dadurch zu einem großen Ganzen zusammenfügen“ (Klemm 1936: 1668). An anderer Stelle hieß es lobend, dass „dieses Erläuterungswerk […] kein Kommentar im alten Sinne“ (Schmidt-Klevenow 1936a: 511) sei. Worin aber trat im Umkehrschluss das „Neue“ dieser Auslegung zutage?

## Die Verschränkung von Medizin und Recht und die „Dehnung“ des Ehegesundheitsgesetzes durch die Erläuterung von Gütt, Linden und Maßfeller

Wie breit die Kluft war, die der Gütt/Linden/Maßfeller als Mittler zwischen den völlig unterschiedlichen Disziplinen Medizin und Jurisprudenz zu überbrücken hatte, zeigt sich in seiner Auslegung des § 1 Abs. 1 Buchst. b EGG: Die Bestimmung hatte das Eheverbot wegen Entmündigung oder Stellung unter vorläufige Vormundschaft zum Gegenstand. Ihre Erläuterung beginnt mit der Wiedergabe des Katalogs möglicher Gründe für eine Entmündigung gemäß § 6 BGB. Im Anschluss an diese Aufzählung konstatieren die Kommentatoren beinahe überrascht: „Es sind also für die Entmündigung nicht lediglich medizinische, sondern auch soziale Gesichtspunkte maßgebend“ (GLM: 61).

Aus rechtswissenschaftlicher Sicht dürfte diese Feststellung eine Selbstverständlichkeit gewesen sein: Niemand konnte allein wegen eines Krankheitsbefundes juristisch belangt werden, sondern allenfalls weil er deshalb andere in ihren Rechten berührte und/oder seine eigenen Rechte nicht wahrnehmen konnte. Die an der Anwendung des Gesetzes beteiligten Mediziner hingegen hatten ihre Behandlungen bis dato immer an vorgeblich eindeutigen Krankheitsbefunden ausgerichtet, die auf der Grundlage vermeintlich empirischer Diagnoseverfahren gestellt wurden. Für sie waren Abwägungen wie die, ob jemand „seine Angelegenheiten nicht zu besorgen vermag“ (§ 6 BGB, zit. nach GLM: 61), Neuland.

Der Herausforderung, Medizinern die Mitwirkung an der Durchsetzung des EGG zu ermöglichen, begegnete der Kommentar mit jener Technik, die 1934 Gütt, Rüdin und Ruttke in ihrer Erläuterung zum GzVeN erstmals erprobt hatten (Yurdakul 2017: 119–132): Genuin rechtswissenschaftliche Fragestellungen wurden mittels medizinischer und eugenischer Argumentationen „aufgelöst“. Die wohl eingängigsten Beispiele für diese Einflussnahme der medizinischen auf die rechtliche Sphäre stellen die Auflistungen von Diagnosen (inklusive Krankheitsbeschreibungen) dar, die Gütt, Linden und Maßfeller den einzelnen Eheverbotsbestimmungen zuordneten (GLM: 47–88). Anders als das GzVeN enthielt das EGG selbst keinen Indikationenkatalog.^6^ Dieser wurde im Gütt/Linden/Maßfeller in Form der besagten Auflistungen quasi nachgeliefert – das Buch führte zu jeder Bestimmung eine Reihe an Diagnosen auf, die nach Ansicht der Autoren darunter fielen. Das hatte eine inhaltliche Verschiebung zur Folge: Wo etwa im Falle des § 1 Abs. 1 Buchst. c ehedem der Fokus auf der juristischen Frage gelegen hätte, was sich hinter dem Terminus „für die Volksgemeinschaft unerwünscht“ verbarg, wurden von den Autoren verstärkt medizinische Befunde in den Blick genommen. Diesen Befunden, so die Suggestion, war eine „volksgemeinschaftliche Unerwünschtheit“ inhärent, sodass es keiner gesonderten Prüfung dieses Tatbestandsmerkmals mehr bedurfte. So handelt der Kommentar die „sozial unerwünschte Ehe“ in einem nur neunzeiligen Unterpunkt (GLM: 65) ab, um anschließend zu betonen: „Das Hauptgewicht bei jeder Ehebeurteilung ist aber auf die Erbbeschaffenheit der Kinder und ihre voraussichtliche Aufzuchts- und Erziehungsmöglichkeit zu legen“ (GLM: 65).

Medizinische Gerichtsexpertisen waren an sich nichts Neues (Geisthövel & Hess 2017: 11f.), der Kommentar räumte dem ärztlichen Wissen in den Verfahren jedoch einen neuen Status ein. Es bedurfte nicht mehr der Transferleistung, die medizinischen Darlegungen dahingehend in den Blick zu nehmen, ob aus ihnen hervorging, dass auch bestimmte Tatbestandsmerkmale oder Tatbestände im juristischen Sinne erfüllt waren. Schließlich knüpften sich nach der Auslegung der Kommentatoren die Rechtsfolgen faktisch gar nicht mehr an solche rechtlichen Tatbestände, sondern direkt an die medizinischen Befunde. Das Verhältnis von juristischer und medizinischer Einschätzung drehte sich damit um 180 Grad: Wo vorher rechtliche Tatbestände entscheidend und medizinische Diagnosen bestenfalls Indikatoren für deren Vorliegen gewesen waren, wurde nun bei Vorliegen bestimmter Diagnosen automatisch ein Tatbestand vorausgesetzt. So ließen sich die rechtlichen Fragen quasi überspringen. Die andere „Qualität“, die Gütt, Linden und Maßfeller ärztlichen Einschätzungen mit dieser Auslegung zusprachen, bereitete den Medizinern den Weg von Sachverständigen zu Richtern.

Einer der Befunde, die die Kommentatoren unter den geistigen Störungen subsumierten, „die eine Ehe für die Volksgemeinschaft unerwünscht erscheinen lassen“ (§ 1 Abs. 1 Buchst. c EGG), war die Psychopathie. Über diesen psychiatrischen Terminus schrieben sie einleitend: „Von einzelnen Forschern wird die Psychopathie gegenüber der Geistesstörung scharf abgegrenzt“ (GLM: 67). Folgt man dem Gesetzeswortlaut, hätte demnach die Diagnose „Psychopathie“ ein Eheverbot auf Grundlage des § 1 Abs. 1 Buchst. c EGG nicht gerechtfertigt – dessen Haupttatbestandsmerkmal war ja gerade das Vorliegen einer geistigen Störung. Erst die Verschränkung der medizinischen mit der rechtlichen Sphäre, die in einer Umdeutung des medizinischen Psychopathiebegriffs zum Rechtsbegriff bestand, erweiterte die Anwendbarkeit der Bestimmung auch auf (sogenannte) Psychopathen: „Gegenüber der Mannigfaltigkeit der Begriffsbestimmungen und -abgrenzungen erscheint es unzweckmäßig, eine neue Begriffsabgrenzung im Sinne der vom Gesetz gemeinten ‚geistigen Störung‘ zu geben“ (GLM: 67). Es bestehe daher „uneingeschränkt die Möglichkeit, daß jeder psychiatrische Facharzt […] Formen der Psychopathie den geistigen Störungen zurechnet, sofern sie derart sind, daß die Ehe des Psychopathen für die Volksgemeinschaft voraussichtlich unerwünscht sein wird“ (GLM: 67).

Es wird deutlich, dass die Einflussnahme der medizinischen auf die rechtliche Sphäre nicht von der Einflussnahme der rechtlichen auf die medizinische Sphäre zu trennen ist: Einerseits ließ der Kommentar medizinische Diagnosen in das Rechtswesen einfließen, andererseits wirkte ebendiese Umdeutung des vormals nur medizinischen Terminus „Psychopathie“ zum Rechtsterminus auf dessen psychiatrische Ursprungsbedeutung zurück. Weil sich das Diktum der Unhinterfragbarkeit des neuen „lebensgesetzlichen Rechts“ auf der Kongruenz von vermeintlich wissenschaftlich objektiven Fach- und Rechtsbegriffen begründete, veränderte die Konversion des medizinischen Befunds zur Voraussetzung eines Tatbestandsmerkmals auch den medizinischen Befund selbst: Nach Erscheinen des Gütt/Linden/Maßfeller war die Psychopathie, von der die Kommentatoren ja selbst einräumen, dass sie in der Psychiatrie vormals „gegenüber der Geistesstörung scharf abgegrenzt“ (GLM: 67) wurde, nicht nur im rechtlichen, sondern handstreichartig auch im medizinischen Sinne zur geistigen Störung erklärt worden. In diesem Zusammenhang muss der amtliche Charakter des Gütt/Linden/Maßfeller hervorgehoben werden: Nicht umsonst war er für alle Ärzte des Reiches eine Pflichtanschaffung. Da sich das Vorliegen der Tatbestände des § 1 Abs. 1 EGG an medizinische Befunde knüpfte, war der Kommentar nicht nur für direkt an der Gesetzesanwendung beteiligte Ärzte verbindlich, sondern für alle diagnostisch tätigen Mediziner.

Die Umdeutung medizinischer Begriffe zu juristischen Termini stellt allerdings nur eine Form der wechselseitigen Einflussnahme beider Sphären aufeinander dar. Neben dieser Einflussnahme auf der begrifflichen Ebene lässt sich eine solche auch auf der methodischen Ebene ausmachen. Eine diesbezügliche Beeinflussung des Rechtswesens durch die Medizin bestand etwa darin, dass Verfahren, die vormals allein dem Erkenntnisgewinn in der medizinischen Forschung und Befunderhebung dienten, im Gütt/Linden/Maßfeller zu Techniken der juristischen Beweisfindung erklärt wurden. Hiermit ist nicht gemeint, dass medizinische Gutachten – wie sowohl vor 1933 als auch nach 1945 ebenfalls (Bauer 2005) – Beweiskraft entfalten konnten. Was Gütt, Linden und Maßfeller vorschwebte, war vielmehr ein Ersatz althergebrachter Methoden rechtswissenschaftlicher Beweisfindung und -erhebung durch diagnostische Verfahren. So schrieben die Autoren über den Befund „angeborener Schwachsinn“, eine Aussage zu dessen Vorliegen könne „oft genug nur unter Berücksichtigung des Sippenbildes getroffen werden“ (GLM: 80). Nach der Vorstellung der Kommentatoren sollte die medizinische Begutachtung der gesamten Familie eines eventuell Erbkranken es ermöglichen, bis dato geltende rechtsstaatliche Prinzipien auszuhebeln: „Überall dort, wo in einem Zweifelsfall in der nächsten Verwandtschaft ein klarer Fall von angeborenem Schwachsinn auftaucht, oder wo mehrere unklare Fälle zusammentreffen […], kann oft der eine Fall die Diagnose des anderen bestätigen“ (GLM: 80).

Dies umschreibt letztlich die in der medizinischen Diagnostik übliche Familienanamnese. Der Quantensprung bestand jedoch darin, dass die Verfasser diese medizinische Technik der Befunderhebung anstelle der juristischen Beweiserhebung treten ließen: Im herkömmlichen Rechtswesen hätte ein Zweifelsfall eine Einstellung des Verfahrens zur Folge gehabt.^7^ Die Erkrankung eines Verwandten wäre anders als in der Medizin, in der ihre Berücksichtigung bei der Diagnosestellung im Interesse des Patienten durchaus Berechtigung hat, kaum beweiskräftig gewesen. Unterdessen ermöglichte die medizinische Herangehensweise des Gütt/Linden/Maßfeller an die an sich juristische Frage, wie *in dubio* zu verfahren sei, sogar, zwei unklare Befunde so in Beziehung zueinander zu setzen, dass sie sich gegenseitig zu vorgeblich eindeutigen Diagnosen ergänzten.

Quasi die umgekehrte Argumentationsweise – hier als „Einflussnahme der rechtlichen auf die wissenschaftliche Sphäre“ umschrieben – findet sich gleichfalls in der Erläuterung zu § 1 Abs. 1 Buchst. d EGG. Die Kommentierung der Bestimmung enthält im Anschluss an die Wiedergabe des Indikationenkatalogs von § 1 Abs. 2 GzVeN folgenden Passus: „Im Rahmen des Ges. zur Verhütung erbkranken Nachwuchses ist der schwere Alkoholismus den in § 1 Abs. 2 des Gesetzes genannten Krankheiten gleichgestellt, macht also auch eheuntauglich“ (GLM: 76).

Dem Wortlaut des Gesetzes zufolge wäre für die Beantwortung der Frage, ob § 1 Abs. 1 Buchst. d auf „schwer Alkoholkranke“ anwendbar war, einzig entscheidend gewesen, ob es sich bei „schwerem Alkoholismus“ medizinisch betrachtet um eine Erbkrankheit handelt. Daran, dass dem nicht so ist, lässt nicht nur die Medizin keinen Zweifel. Auch das GzVeN selbst und seine Verordnungen machen deutlich, dass Alkoholismus keine Erbkrankheit ist, auch nicht „im Sinne des Gesetzes“. So wurde er nicht unter den „Erbkrankheiten“ in § 1 Abs. 2 GzVeN aufgeführt; die Sterilisation aufgrund dieses Leidens wurde in einer getrennten Norm (§ 1 Abs. 3 GzVeN) geregelt. Dezidiert differenziert Artikel 4 Abs. 2 der Zweiten Ausführungsverordnung zum GzVeN, ein Betroffener sei entweder „Erbkranker oder Alkoholiker“ (Reichsministerium des Innern 1934b: 476).

Nur über das juristische Konstrukt der (rechtlichen) Gleichstellung vermochten es Gütt, Linden und Maßfeller, Alkoholismus zur Erbkrankheit umzudeklarieren, womit er unter § 1 Abs. 1 Buchst. d EGG fiel. Da diese Sichtweise unmittelbar auf die Medizin und Recht verflechtende Auslegungsmethodik zurückzuführen ist, verwundert nicht, dass konventionellere Kommentierungen sie nicht teilten. So erläuterten Stuckart und Globke, Alkoholismus sei „[k]eine eigentliche Erbkrankheit“ (Stuckart & Globke 1936: 171). Sie schlugen vor, Alkoholismus den volksgemeinschaftlich unerwünschten geistigen Störungen zuzurechnen, sodass „das Ehehindernis des § 1 Abs. 1 Buchst. c vorliege[…]“ (Stuckart & Globke 1936: 171). Zwar plädierten sowohl Stuckart und Globke als auch Gütt, Linden und Maßfeller für ein Eheverbot für Alkoholkranke. Der Vergleich der Erläuterungen dient jedoch nicht dazu, festzustellen, ob eine der Schriften eine extensivere Auslegung praktizierte. Er zeigt vielmehr, dass sich beide unterschiedlicher Auslegungstechniken bedienten.

Darüber, dass sich die Methodik von Gütt, Linden und Maßfeller durchsetzte, gibt die Rechtsprechung Aufschluss: Alkoholabhängigen wurde das Ehetauglichkeitszeugnis regelmäßig gemäß § 1 Abs. 1 Buchst. d EGG verweigert (Gründler 1943: 6f.).

Generell ist festzuhalten: Alle Einflussnahmen von wissenschaftlicher und rechtlicher Sphäre aufeinander im Gütt/Linden/Maßfeller leisteten einer Dehnung des Anwendungsbereichs der kommentierten Paragrafen Vorschub. Auch in dieser Hinsicht folgte er dem „Vorbild“ des Gütt/Rüdin/Ruttke (Yurdakul 2017: 119–124). Beide Kommentare beförderten im „Erbgesundheitsrecht“ letztlich einen Trend, den die NS-Rechtsgeschichte für nahezu alle (Teil‑)Gebiete des Rechts konstatiert und den Bernd Rüthers für das Privatrecht mit der Formel der „unbegrenzten Auslegung“ umschrieben hat (Rüthers 2017).

## Die Medizin als Ressource für das Recht

Die Dehnung des Gesetzes war nicht das einzige Resultat der Verschränkung von Wissenschaft und Recht im Kommentar: Durch die Einflussnahme der medizinischen auf die rechtliche Sphäre wurde die Medizin zur Ressource für die Jurisprudenz. Im Umkehrschluss ließ die Einflussnahme der rechtlichen auf die medizinische Sphäre die Rechtswissenschaft zur Ressource für die Medizin werden (Yurdakul 2017: 108).

Die erwähnte Suche nach dem „*einen* richtigen, wesensentsprechenden Begriff“ (Schröder 2016: 45) mündete nicht nur in eine Mobilisierung der Medizin als Ressource für die Jurisprudenz, die darin bestand, dass der Kommentar medizinische Fachbegriffe zu Rechtsbegriffen umdeutete, die in ihrer Bedeutung dezidiert mit dem medizinischen Sinngehalt übereinstimmten und deshalb als wissenschaftlich fundiert und nicht mehr hinterfragbar galten. Sie wirkte sich auf den gesamten Prozess der Rechtsfindung aus. Naturgemäß ließ sich nicht mehr allein mittels juristischer Techniken prüfen, ob ein solcher Begriff auf einen bestimmten Fall anwendbar war. So war es im Fall des EGG nur noch unter Beteiligung von Medizinern möglich, Entscheidungen auf Grundlage dieser Vorschrift zu treffen. Festzuhalten ist, dass es der Kommentar war, der die Übernahme von Methoden der medizinischen Befunderhebung in das juristische Verfahren forderte. Zugleich betonte er deren legitimierenden Charakter für die Rechtsprechung. In Abgrenzung gegen die als „subjektiv“ verbrämte juristische Herangehensweise, die in erster Linie in der Befragung des Delinquenten bestand, hoben die Autoren die vorgebliche Objektivität der medizinischen Diagnosestellung hervor:

Wenn auch gute Menschenbeobachtung vielfach ein Urteil über die Glaubwürdigkeit einer verneinenden Auskunft ermöglichen kann, so liegen die Beweggründe für eine Verheimlichung doch allzu nahe. Die Erfassung der Geschlechtskranken unter den Heiratskandidaten arbeitet daher um so [sic!] zuverlässiger, je mehr sie die subjektiven Feststellungen durch objektive ergänzt (GLM: 51).

Diese Form der Ressourcenmobilisierung machte quasi die Medizin als Disziplin insgesamt zur Ressource für die Rechtswissenschaft als solcher, indem sie sie überhaupt erst mit dem „lebensgesetzlichen“ NS-Rechtsdenken kompatibel machte. Daraus ergab sich eine weitere Ressourcenmobilisierung für die Jurisprudenz auf anderer Ebene: Die Medizin ebnete dem Rechtswesen den Weg in Lebensbereiche der „Volksgenossen“, auf die es in der liberalen Normenstaatlichkeit der Weimarer Republik keinen Zugriff gehabt hatte. Auch hierbei spielte der Kommentar die entscheidende Rolle. Beispiele finden sich wiederum vor allem in dessen Auslegung zu § 1 Abs. 1 Buchst. c EGG. Hier führen die Autoren in einem Abschnitt über *Senile Störungen* an:

Es gibt eine Reihe von psychischen Störungen des hohen Lebensalters, die man wohl als umweltbedingte bzw. erworbene ansprechen kann. Die Fälle, in denen derartige Personen verhältnismäßig junge Frauen zu heiraten beabsichtigen, sind nicht gerade selten. Solche Ehen sind aber rassenhygienisch überaus unerwünscht. Hier wird das Vorliegen einer präsenilen geistigen Störung stets zur Grundlage des Eheverbotes zu machen sein (GLM: 74).

Für die thematisierte Fragestellung spielt weniger die Offenheit eine Rolle, mit der Gütt, Linden und Maßfeller einer willkürlichen Anwendung des Gesetzes das Wort redeten. Entscheidender ist, dass sie aufgrund einer (wenn auch höchst unkonkreten) medizinischen Diagnose die Einflusssphäre der Justiz auf einen Bereich des Privatlebens ausdehnten, der vormals vor staatlichen Eingriffen geschützt gewesen war.

Die Kommentierung wirkte auf etwas hin, das – in Anlehnung an Alf Lüdtke (1994: 188) und Jürgen Kocka (1994), die für die DDR-Geschichtsschreibung den Begriff einer „Durchherrschung“ der Gesellschaft prägten (Lüdtke 1994: 188; Kocka 1994), sowie Rüdiger Hachtmann (2011: 47–51), der diesen Terminus um das Attribut „kumulativ“ ergänzt auf die NS-Geschichte angewandt hat – als „juristische Durchherrschung“ der Gesellschaft charakterisiert werden könnte: Laut dem Gütt/Linden/Maßfeller sollte die Justiz die durch den Zweck der rassenhygienischen „Aufartung“ geheiligten Mittel erhalten, bis in die „feinsten Verästelungen“ (Kocka 1994: 548) der Privatsphäre vorzudringen.

Die Praxis der Erbgesundheitsgerichte orientierte sich an der Forderung der Juristischen Wochenschrift, es dürfe „in Zukunft kein Urteil mehr geben, das sich im Eheprozeß mit Gesundheitsfragen beschäftigt und sich nicht mit den Ansichten von G ü t t , L i n d e n und M a ß f e l l e r [Hervorhebung im Original] auseinandersetzt“ (Meinhof 1936: 3446). „Auseinandersetzung“ bedeutete für die Richter, dem Hinweis der Rezension zu folgen, der Kommentar lasse „eine Fortbildung [in der Praxis] zu, wie sie jedes lebendige Recht braucht“ (Meinhof 1936: 3446). Indem sie auch Ansichten, die sich aus der Erläuterung nur implizit herauslesen ließen, in ständiger Rechtsprechung zementierten, gelang es den Tribunalen, der Auslegung Gütts, Lindens und Maßfellers im selben Atemzug zu folgen, in dem sie sie noch „übertrafen“. So sprachen weder Gesetz noch Kommentar davon, Ehetauglichkeitszeugnisse „endgültig“ (Gründler 1943: 4, 8f.) zu versagen. Genau dies geschah jedoch regelmäßig, in Frankfurt am Main bis Ende 1942 zum Beispiel in 124 Fällen (Gründler 1943: 9). Die Erbgesundheitsrichter antizipierten mit dem Konstrukt der „endgültigen Versagung“, was die Autoren der Erläuterungsschrift bereits unter Berufung auf medizinische Erwägungen insinuierten: Sie behaupteten für eine große Zahl von Erkrankungen, wenn diese festgestellt würden, sei die Eheschließung „in der Regel“ (GLM: 72), „[a]uf alle Fälle“ (GLM: 72) oder „stets“ (GLM: 74) zu untersagen. Zugleich postulierten sie, diese Krankheiten seien „erbmäßig-bedingt[…]“ (GLM: 69) – mithin nicht heilbar. Das legte die Aussprache lebenslanger Eheverbote nahe, ohne explizit zu werden. Der Kommentar stellte ergo auch eine Art „Vehikel zur weiteren Selbstermächtigung“ dar.

Diese Mobilisierungen von Ressourcen auf Ebene der unmittelbaren justiziellen Kompetenzen sind nicht von jener übergeordneten Ressourcenmobilisierung zu trennen, die in der Relegitimation der Rechtswissenschaft unter nationalsozialistischen Vorzeichen mittels der Medizin bestand. Die Möglichkeit, nahezu unumschränkt in das Leben des Individuums einzugreifen, war vielmehr gleichfalls Voraussetzung dafür, dass das Rechtswesen den Anforderungen des NS-Staates genügte. Sie stellt eines der wesentlichen Charakteristika des Maßnahmenstaates, wie Ernst Fraenkel ihn beschrieben hat, dar (Fraenkel 1974: 88f.). Es handelt sich demnach um eine Ressourcenmobilisierung in doppelter Hinsicht: Zur Entgrenzung des Rahmens selbst, in dem menschliches Handeln juristischer Sanktionierung unterworfen werden konnte, kommt hinzu, dass in den Augen des NS-Regimes ebendiese Entgrenzung der Justiz erst zu ihrer Legitimation im autoritären Staat verhalf.

## Das Recht als Ressource für die Medizin

Spiegelbildlich ergänzt wurden die Mobilisierungen von Ressourcen für die Jurisprudenz und Justiz von Ressourcenmobilisierungen für die Medizin auf unterschiedlichen Ebenen: Mit der „juristischen Durchherrschung“, die der Kommentar propagierte, ging die Vorstellung einer „medizinischen Durchherrschung“ der Gesellschaft einher. Wo die Kommentatoren die Erweiterung justizieller Spielräume mit medizinischen Erwägungen begründeten, ebneten sie im Umkehrschluss einer technokratischen Durchplanung der Gesellschaft nach medizinisch-biologischen Kriterien mittels genuin rechtswissenschaftlicher Techniken der Gesetzesauslegung juristisch den Weg: „Wenn auch der Wortlaut des Gesetzes nur von der Möglichkeit spricht, daß einer der Verlobten an einer geistigen Störung im Sinne des Gesetzes leidet, so ist doch mit Rücksicht darauf, daß das Gesetz eine zu schließende Ehe betrifft, der andere Verlobte wesentlich mit zu berücksichtigen“ (GLM: 65).

Nicht erbbiologische Überlegungen sind es, die Gütt, Linden und Maßfeller anführen, um die „sozialpolitische Einflußnahme und Kontrolle von Experten auf die soziale und sexuelle Geschlechtergemeinschaft Ehe“ (Czarnowski 1991: 15) zu rechtfertigen. Stattdessen argumentieren die Autoren in juristischen Auslegungskategorien, namentlich mit Gesetzeswortlaut und „Intention des Gesetzgebers“. Über die rechtswissenschaftliche Herleitung eröffneten die Kommentatoren der Medizin die Perspektive, über Wert und Legitimität jeder Ehe im Vorfeld nach wissenschaftlichen Maßstäben zu befinden:

Das bedeutet, daß z. B. eine verhältnismäßig geringe geistige Abartigkeit, wie etwa psychopathische Gemütlosigkeit, die als solche den Träger dieser Eigenschaft noch nicht unbedingt als volksgemeinschaftlich unerwünscht erscheinen läßt, seine Ehe unerwünscht machen kann, sofern er einen ebenso gearteten Verlobten gewählt hat (GLM: 65).

Dass jede Eheschließung unter dem Vorbehalt der „medizinisch-biologischen Kompatibilität“ der Partner stand, erhöhte den Stellenwert von Medizin und Biologie in der Wissenschaftslandschaft insgesamt. Dieser Befund korrespondiert mit der Feststellung, dass generell „[i]n keinem politischen System […] eugenischen Postulaten und Programmen jemals eine derart hohe Priorität eingeräumt worden [war] wie ab 1933 im nationalsozialistischen Deutschland“ (Schmuhl 2008: 102). Diese Aufwertung war mit konkreten personellen und finanziellen Ressourcenzuwächsen verbunden.

Von dem Umfang, in dem bereits die Vorschrift selbst Ressourcen für die Medizin mobilisierte, zeugt § 8 Abs. 2 EGG. Wie dargelegt, verschob er das Inkrafttreten des § 2, nach dem die Vorlage eines Ehetauglichkeitszeugnisses vor jeder Hochzeit obligatorisch sein sollte, auf einen vom Reichsinnenminister zu bestimmenden Zeitpunkt. Die Begründung zu dem Gesetz führte dies darauf zurück, dass „es nicht möglich sein wird, den Gesundheitsämtern sofort geeignete Ärzte und Hilfspersonal für die Aufgaben in genügender Zahl zur Verfügung zu stellen“ (zit. nach GLM: 39). Tatsächlich schossen die Zahlen der Medizinstudenten nach Inkrafttreten des Gesetzes an vielen Fakultäten nach oben, sehr signifikant etwa in Freiburg (Eiberg et al. 2002: 222). Dass § 2 letztlich nie vollumfänglich in Kraft gesetzt wurde, zeigt aber auch die politischen Grenzen der Ressourcenmobilisierung für die Medizin: Die Norm hätte die Mediziner zu Richtern über alle (heiratswilligen) „Volksgenossen“ gemacht und sie dem politischen Einfluss entzogen. Die NS-Machthaber wollten sich jedoch die „staatliche[…] Kontrollmöglichkeit“ (Czarnowski 1991: 179; ähnlich Bock 1986: 103) unter allen Umständen bewahren.

Der kurz nach Verabschiedung des Gesetzes erschienene Gütt/Linden/Maßfeller ging noch von einer Inkraftsetzung des § 2 aus und warb für eine Steigerung der Zahl der reichsdeutschen Ärzte, die weit über das hinausging, was sich aus der Begründung des Gesetzes ableiten ließ. Dort war nur von weiterem Personal für die Gesundheitsämter die Rede. In vielen Beschreibungen von Befunden, bei deren Vorliegen das Ehetauglichkeitszeugnis verweigert werden sollte, mahnten die Autoren, in Zweifelsfällen – mitunter sogar in jedem Fall – Fachärzte zurate zu ziehen. Dies implizierte einen enormen zusätzlichen Bedarf an Medizinern. So sah die von dem Kommentar propagierte Praxis etwa vor, dass selbst Verlobte mit Erkältungsanzeichen sich einer Untersuchung durch einen Lungenfacharzt zu unterziehen hatten. Denn selbst „[h]inter harmlosen Erscheinungen“ könne sich eine Tuberkulose verbergen, die allein „durch einen in der Tuberkulosediagnostik geübten und erfahrenen Arzt“ (GLM: 49) auszuschließen sei.

Hinzu kam, dass die Verfasser aus dem EGG medizinwissenschaftliche Themenfelder ableiteten, die sie im Interesse einer möglichst weitgehenden Anwendung der Vorschrift stärker beforscht sehen wollten. So ist einerseits davon auszugehen, dass bereits die Aufzählung konkreter Diagnosen, die sich ja weder in dem Gesetz und seinen Verordnungen selbst fanden, noch in anderen Kommentierungen,^8^ die Forschung beeinflusste. Schließlich waren Studien zu den Indikationen, die die Schrift von dem Gesetz erfasst sah, nicht mehr nur für eine medizinische Fachöffentlichkeit interessant, sondern versprachen, breiter rezipiert zu werden.

Damit zusammenhängend formulierten Gütt, Linden und Maßfeller andererseits „Forschungsaufträge“. Über die ihrer Meinung nach ein Eheverbot gemäß § 1 Abs. 1 Buchst. a EGG begründende Gonorrhoe schrieben die Kommentatoren etwa:

Besonders bei der Frau ist die Feststellung des Trippers, zumal in seiner chronischen Form, selbst für den Arzt schwierig. Wenn es gelänge, alle Tripperkranken von der Eingehung der Ehe abzuhalten, so könnte hierdurch in Deutschland eine erhebliche Zahl heute ausfallender Geburten erhalten bleiben (GLM: 58).

In der *Dermatologischen Wochenschrift *vom 20. August 1938 erschien daraufhin ein Artikel des Stabsarztes Wilhelm Burger unter der Überschrift *Zur Erfassung der Ansteckungsquellen und zur Frage der Feststellung der Heilung beim Tripper des Weibes* (Burger 1938). Einleitend zitiert der Verfasser drei längere Stellen aus dem Abschnitt des „amtliche[n] Kommentar[s] von GÜTT, LINDEN, MASSFELLER [Hervorhebung im Original]“ (Burger 1938: 1006) zur Gonorrhoe-Erkrankung. Die „Richtlinien“ (Burger 1938: 1006), die der Kommentar aufstellt, um die Ansteckungsgefahr im Fall eines Tripper-Leidens zu beurteilen, dienen als Aufhänger von Burgers Studie. Er betont, dass das von Gütt, Linden und Maßfeller aufgestellte Kriterium mindestens eines negativen Provokationstests zum Ausschluss einer Ansteckungsgefahr seine Untersuchung veranlasst hat: „Die vorliegende Arbeit zeigt wiederum den mit dieser Regelung erzielten Fortschritt und gibt andererseits auch einen Hinweis darauf, wie häufig es angebracht sein kann, das vorgeschriebene Mindestmaß zu überschreiten“ (Burger 1938: 1006). Allein dieses Beispiel zeigt, wie beträchtlich die Ressourcen waren, die der Kommentar für die Forschung mobilisierte: Für seine Untersuchung erhielt Burger Kapazitäten, um „das Krankengut der Klinik [Würzburg] vom Jahre 1930–1937“ (Burger 1938: 1009) aufzubereiten. Hierbei handelte es sich um die Patientenakten von insgesamt 422 an Gonorrhoe erkrankten Frauen (Burger 1938: 1009).

Burgers Text war kein Einzelfall, wenngleich er besonders anschaulich zeigt, wie die Schrift von Gütt, Linden und Maßfeller der Medizin Impulse gab. Vom Einfluss der Erläuterung auf die medizinische Forschung zeugt eine Vielzahl weiterer Fachartikel, die sie in ihren Literaturverzeichnissen aufführten oder direkt aus ihr zitierten.^9^

Wie sehr die stärkere Orientierung an medizinischen Fragen die Kommentierung des EGG von „klassischen“ Gesetzeserläuterungen unterschied, zeigt der Vergleich mit der eher konventionell-juristischen Auslegung des Blutschutzgesetzes im gleichen Buch.

## Die Erläuterung des Blutschutzgesetzes durch Gütt, Linden und Maßfeller

Bereits ein Vergleich der von Gütt, Linden und Maßfeller für ihre Erläuterung des EGG gewählten Zwischenüberschriften mit denen des Abschnitts zum Blutschutzgesetz lässt den Schluss zu, dass die Kommentierung beider Vorschriften in unterschiedlichem Stil erfolgte. So wurde die Auslegung des § 1 EGG durch Zwischentitel wie *Allgemeine Übersicht über die in Betracht kommenden Krankheiten* (GLM: 47), *Die Geschlechtskrankheiten im Besonderen* (GLM: 51) und, innerhalb des letztgenannten Teilkapitels, durch Unterüberschriften wie *Syphilis *(GLM: 54) und so fort untergliedert. Indes sind die Abschnitte der Erörterung des § 1 Blutschutzgesetz mit Überschriften und Zwischentiteln versehen, die einer eher juristischen Diktion folgen. Sie lauten etwa: *Begriffsbestimmungen* (GLM: 195), *Die Eheverbote wegen jüdischen Bluteinschlags* (GLM: 201), *Materielles Eheschließungsrecht* (GLM: 201) und *Die Rechtsfolgen der Übertretung der Eheverbote* (GLM: 205).

Dies fiel auch Rezensenten auf. So hob Robert Gaupp den Kontrast hervor, indem er mit Blick auf die Auslegung des EGG feststellte, „dieser Teil des Buches [sei] für den Arzt von besonderem Wert“ (Gaupp 1937: 209). Unterdessen würden in der Erläuterung des Blutschutzgesetzes „naturgemäß mehr rechtliche als ärztliche Probleme erörtert“ (Gaupp 1937: 209).

Woran lassen sich die Unterschiede in der Erläuterung beider Gesetze festmachen? Die zentralen Begriffe, mit denen das Blutschutzgesetz und seine Erste Ausführungsverordnung operierten – „Jude“ und „jüdischer Mischling“ –, waren als reine Rechtsbegriffe angelegt und in ihrer Bedeutung nicht kongruent mit den gleichlautenden rassentheoretischen Begriffen (Essner 2002: 161, 171). § 1 Abs. 2 und 3 der Ersten Ausführungsverordnung zum Blutschutzgesetz (Reichsministerium des Innern 1935b, im Folgenden paragraphengenau zitiert als „Erste AusfVO z. Blutschutzgesetz“) verwiesen bezüglich der Definitionen beider Begriffe auf die Erste Verordnung zum Reichsbürgergesetz vom 14. November 1935 (Reichsministerium des Innern 1935a, im Folgenden paragraphengenau zitiert als „Erste VO z. Reichsbürgergesetz“). Demnach war Jude, „wer von mindestens drei der Rasse nach volljüdischen Großeltern“ (§ 5 Abs. 1 Erste VO z. Reichsbürgergesetz) und jüdischer Mischling, „wer von einem oder zwei der Rasse nach volljüdischen Großeltern abstammt“ (§ 2 Abs. 2 Erste VO z. Reichsbürgergesetz). „Mischlinge“, die von zwei jüdischen Großeltern abstammten, galten jedoch ebenso als „Volljuden“, wenn sie der jüdischen Religion angehörten, mit einem Juden verheiratet waren, einer nach dem 15. September 1935 geschlossenen Ehe mit einem jüdischen Partner entstammten oder aus einer außerehelichen Beziehung mit einem Juden hervorgegangen und nach dem 31. Juli 1936 geboren waren (§ 5 Abs. 2 Buchst. a, b, c, d Erste VO z. Reichsbürgergesetz). Die Erste Verordnung zum Blutschutzgesetz ergänzte die Kategorisierung in „Volljuden“ und „Mischlinge“, indem sie die „Mischlinge“ binnendifferenzierte – in „Mischlinge mit zwei volljüdischen Großeltern“ (§ 3 Abs. 1 Erste AusfVO z. Blutschutzgesetz) („Mischlinge ersten Grades“) und „Mischlinge[…], die nur einen volljüdischen Großelternteil haben“ (§ 2 Erste AusfVO z. Blutschutzgesetz) („Mischlinge zweiten Grades“). Gemäß §§ 2, 3 und 4 der Ersten Ausführungsverordnung zum Blutschutzgesetz durften „Mischlinge ersten Grades“ nur Juden im Sinne des Gesetzes oder andere „Mischlinge mit zwei volljüdischen Großeltern“ heiraten. „Mischlinge zweiten Grades“ durften nur „Deutschblütige“ heiraten. Dies sollte den Kommentatoren zufolge dazu führen, „daß mit der Zeit die Rassenmischlinge verschwinden“ (GLM: 222).

Dem „Forschungsstand“ der Rassentheorie entsprach all das nicht. Das Kind eines „Mischlings zweiten Grades“ und einer „Deutschblütigen“ war nur juristisch „deutschblütig“, da es keinen „volljüdischen“ Großelternteil hatte. Aus rassenbiologischer Sicht hatte es jedoch „jüdische Gene“ (Essner 2002: 162f.). Dieser Widerspruch war auch Gütt, Linden und Maßfeller bewusst. Mit Blick auf das Raster, nach dem Menschen in „Juden“, „Mischlinge“ und „Deutschblütige“ eingeteilt wurden, mussten sie einräumen: „Es ist sicherlich richtig, daß diese Regelung im Einzelfall auch einmal zu biologisch nicht zu rechtfertigenden Entscheidungen führen kann“ (GLM: 198). Auch die Geschichtswissenschaft hat diesen „Widerspruch zwischen dem juristischen Judenbegriff und der aus der NS-Rassenlehre entlehnten Vorstellung der ‚Judenrasse‘“ (Hering Torres 2006: 244) herausgearbeitet (etwa auch bei Schnitzler 2012: 163).

Anders als im Fall des EGG machten Gütt, Linden und Maßfeller in ihrem knapperen Kommentar zum Blutschutzgesetz kaum Anstalten, die juristische und fachspezifische – im Falle des Blutschutzgesetzes: rassentheoretische – Bedeutung des Juden- und des Mischlingsbegriffs in Einklang zu bringen. So verzichteten sie darauf, rassenkundliche Ansichten zu referieren, die zumindest bis zu einem gewissen Grad kompatibel mit der Gesetzgebung waren, obwohl es sie – wenngleich Minderheitsmeinungen – gab (Essner 2002: 162).

Überhaupt sahen die Autoren davon ab, fachwissenschaftliche Texte anzuführen, wie sie es in der Erläuterung des EGG getan hatten: Aus dem Namensregister des Kommentars geht hervor, dass in dem Teil des Buches, der auf das EGG eingeht (GLM: 45–194), an 17 Stellen auf Mediziner verwiesen wird (GLM: 348). Hingegen finden sich in dem Abschnitt zum Blutschutzgesetz (S. 195–244) weder Erwähnungen von Ärzten noch von Rassentheoretikern, sondern nur von Juristen (GLM: 348). Hierzu korrespondiert, dass sich Gütt, Linden und Maßfeller einer erheblich rechtswissenschaftlicheren Herangehensweise bedienten. Wo der Leser der medizinischen Erörterungen der Autoren zum EGG nun rassentheoretische Überlegungen hätte erwarten können, fanden sich stattdessen Ausführungen, die eher an eine herkömmliche juristische Kommentierung denken ließen: Die Frage etwa, wann Eheschließungen zwischen einem „Mischling ersten Grades“ und einem „Mischling zweiten Grades“ oder einem „Deutschblütigen“ ausnahmsweise genehmigt werden sollten, beantwortete der Gütt/Linden/Maßfeller nicht, indem er – analog zu den in der Erläuterung des EGG für die erbgesundheitliche Beurteilung einer Ehe angeführten medizinischen Parametern – rassenbiologische Parameter ins Feld führte. Stattdessen bediente er sich eines Analogieschlusses, also einer althergebrachten Rechtsfigur: Gemäß § 2 Abs. 1 Reichsbürgergesetz (Reichsministerium des Innern 1935f, im Folgenden paragraphengenau zitiert als „Reichsbürgergesetz“) könne auch der „Mischling ersten Grades“ „endgültiger Reichsbürger“ werden, wenn er „durch sein Verhalten beweist, daß er gewillt und geeignet ist, in Treue dem Deutschen Volk und Reich zu dienen“ (§ 2 Abs. 1 Reichsbürgergesetz). Jeder „endgültige Reichsbürger“ habe das Recht, einen „Deutschblütigen“ oder „Mischling zweiten Grades“ zu ehelichen. Daher sei die Genehmigung zur Eheschließung zwischen einem „Mischling ersten Grades“ und einem „Deutschblütigen“ oder „Mischling zweiten Grades“ zu erteilen, wenn der „Mischling ersten Grades“ die Voraussetzungen erfülle, um „endgültiger Reichsbürger“ zu werden (so sinngemäß GLM: 220).

Warum aber brachten Gütt, Linden und Maßfeller ihre Auslegung nicht soweit als möglich mit der Rassenkunde in Einklang? Hierzu bestand schlicht keine Notwendigkeit, denn Rassentheoretiker waren an der Anwendung des Gesetzes so gut wie nicht beteiligt –10 wohl auch, weil die Raster, nach denen sie Menschen klassifizierten, so obskur waren, dass sie sich jeder Kodifizierung entzogen.

Die Prüfung, ob ein „Ehehindernis wegen jüdischen Bluteinschlags“ (GLM: 204) im Sinne des Gesetzes vorlag, oblag dem Standesbeamten beziehungsweise – falls er das Aufgebot versagte und die Verlobten dagegen vorgingen – dem Amtsgericht und dessen übergeordneten Instanzen (GLM: 204). Auch prüften diese Institutionen nicht anhand rassenkundlicher Gutachten. Allein Geburtsurkunden der Verlobten und Geburts- und Heiratsurkunden sowie Taufzeugnisse ihrer Vorfahren wurden herangezogen (GLM: 203f.) – rechtliche Dokumente also. Anders als in Ehegesundheitsangelegenheiten hatten „Fachwissenschaftler“ in Blutschutzangelegenheiten – also Rassentheoretiker – weder als sachkundige Laienrichter noch als anderweitige Entscheidungsinstanzen Einfluss auf die Beschlüsse. Gütt, Linden und Maßfeller kommentierten quasi zielgruppengerecht: Eine systematische Verschränkung von Fachwissenschaft und Recht betrieben sie nur, wo es in der Praxis einen „personellen Anknüpfungspunkt“ an die betreffende Wissenschaft gab.

Freilich fanden sich Anleihen an die Rassenkunde unvermeidlich auch in den Ausführungen Gütts, Lindens und Maßfellers zum Blutschutzgesetz. Dies brachte der Regelungsgegenstand zwangsläufig mit sich.^11^ Eine systematische Verschränkung von Rassenkunde und Recht lässt sich aus den vereinzelt in die Erläuterung eingestreuten rassenkundlichen Befunden aber nicht ableiten. Auch kam es folglich zu keiner wechselseitigen Ressourcenmobilisierung durch den Kommentar. Weder betonten die Autoren den legitimierenden Charakter vorgeblich wissenschaftlich fundierter Befunde der Rassenkunde für das Rechtswesen, noch leiteten sie aus dem Gesetz etwa einen weitergehenden rassenkundlichen Forschungsbedarf ab. Textstellen, an denen Desiderate aufgezeigt wurden oder gefordert wurde, Rassentheoretiker als Gutachter hinzuzuziehen, sucht man nahezu vergebens.^12^ Entsprechend erfuhr der Gütt/Linden/Maßfeller in der rassenbiologischen *scientific community* weit weniger Aufmerksamkeit als in der medizinischen. Die *Zeitschrift für Rassenkunde* widmete ihm nur eine kurze Besprechung, die sich im Wesentlichen auf eine Inhaltsangabe beschränkte (Steinwallner 1937).

Zu betonen ist, dass der Befund, Gütt, Linden und Maßfeller verzichteten auf eine Rassenkunde und Recht verflechtende Kommentierung des Blutschutzgesetzes, keine Rückschlüsse darüber zulässt, wie extensiv sie die Vorschrift auslegten. Die Verfasser kommentierten das Blutschutzgesetz nicht etwa „rechtsstaatlicher“ als das EGG. Sie bedienten sich bei der Erläuterung beider lediglich unterschiedlicher Auslegungsmethodiken.

Hieraus einen „Masterplan“ abzuleiten, der der Arbeitsweise der Kommentatoren zugrunde gelegen hätte, wäre jedoch ein Fehlschluss. Vielmehr scheint die veränderte Technik juristischen Erläuterns schlicht eine Reaktion auf die sich ändernde Gerichtslandschaft nach 1933 gewesen zu sein. Die nationalsozialistischen Bestrebungen, das Kommentarwesen zu reformieren, sollten daher nicht allein vor dem Hintergrund rechtstheoretischer Konzepte des „Dritten Reichs“ betrachtet werden, wie das bislang in der Rechtsgeschichte weitgehend der Fall war (etwa bei Henne 2006: 354; Kästle 2013: 434–437; Kästle-Lamparter 2016: 77–79). Sie müssen mit der konkreten Veränderung der Justizlandschaft zusammengedacht werden, namentlich mit der forcierten Installation Sachverständiger und sachkundiger Laienrichter als Entscheidungsinstanzen.

## Die Kommentierung „neuen Typs“ und die sachkundigen Laienrichter

Die hier untersuchte, Wissenschaften und Recht verschränkende Methodik juristischer Kommentierung war an Gesetze gebunden, auf deren Grundlage sachverständige „Experten“ entschieden. Dieser Befund lässt auf den ersten Blick vermuten, dass die Schriften, die sich dieser neuartigen Auslegungstechnik bedienten, ein unbedeutendes Subgenre innerhalb der Textgattung Gesetzeserläuterung bildeten. Eine solche Einschätzung greift für die Zeit der Hitler-Diktatur aus zwei Gründen zu kurz.

Erstens hat sich die NS-Forschung bislang kaum mit den mit sachkundigen Laien besetzten Gerichten beschäftigt. Dies lässt leicht das tatsächliche Ausmaß übersehen, in dem das Regime sie bis Mitte der 1930er Jahre auf unterschiedlichsten Rechtsgebieten installierte. Zu nennen sind neben den Erbgesundheitsgerichten vor allem die zahlreichen ständischen Gerichte, in denen etwa Publizisten (Bezirksgerichte der Presse), Landwirte (Anerbengerichte) sowie Unternehmer und sogenannte betriebliche Vertrauensleute (soziale Ehrengerichte) als Richter auftraten.

Zweitens besaßen diese neuartigen Spruchkörper einen höheren Stellenwert, als bislang herausgearbeitet wurde. Besonders in den ersten Jahren des „Dritten Reichs“ avisierten NS-Juristen eine Stärkung des sachkundigen Laienrichtertums in mehrfacher Hinsicht (Yurdakul 2020). So ging von dem Ministerialbeamten Josef Schafheutle (1904–1973) 1937 die Initiative aus, Schöffen an Kammern, die mit Verkehrsunfallverfahren befasst waren, durch „sachverständige Volksrichter“ zu ersetzen. Diese sollten „in der Untersuchung und Beurteilung von Verkehrsunfällen besondere Erfahrung haben.“ (zit. nach Lemke-Küch 2014: 122). Andere Stimmen forderten gar, auch juristische Beisitzer in Kollegialgerichten der Zivilgerichtsbarkeit durch sachkundige Laien zu ersetzen (Kross 1991: 209). Einen weiteren Weg, sachkundigen Laien zulasten etablierter Institutionen des Rechtswesens neue Aufgabenfelder zu erschließen, beschritt 1936 – erfolglos – Falk Ruttke: Er regte an, den ordentlichen Gerichten die Zuständigkeit für die Ehescheidung zu entziehen und sie den Erbgesundheitsgerichten zu übertragen (Heller 2015: 202f.). Der Rechtswissenschaftler Leopold Zimmerl (1899–1945) forderte, in der gesamten Justiz nur noch sachverständige Laienrichter zuzulassen (Zimmerl 1934: 23) und sie den juristisch ausgebildeten Vorsitzenden der Kammern in Rechten und Pflichten gleichzustellen (Lemke-Küch 2014: 123). Erstaunlich mutet zunächst an, dass all diese Vorstöße aus der Jurisprudenz selbst kamen und sich seitens der Rechtspraktiker kein Widerstand regte. Der partielle Kompetenzverlust, den die Umsetzung der Planungen für die Juristen bedeutet hätte, wurde jedoch mehr als aufgewogen: Zum einen machte die Autorität, die die vorgeblichen Experten den Gerichtsentscheidungen verleihen sollten, die Justiz überhaupt erst anschlussfähig an die Rechtskonzepte des „Dritten Reichs“. Zum anderen ermöglichten die Sachverständigen dem Rechtswesen, in Bereiche des Privatlebens der „Volksgenossen“ einzudringen, die seiner Einflusssphäre in der Weimarer Zeit entzogen gewesen waren.

Zeitweise war angedacht, sachkundige Laienrichter sogar in der allgemeinen Verwaltungsrechtspflege einzusetzen. Die Initiative scheiterte, weil es misslang, auf diesem Gebiet besonders sachverständige juristische Laien zu finden (Schiffmann 1974: 55f.). Dies verwundert kaum, schließlich sind die Verwaltungswissenschaften historisch aus den Rechtswissenschaften hervorgegangen und eng mit ihnen verwandt (Bogumil & Jann 2005: 28–31). Mithin waren die Juristen selbst auf diesem Gebiet „sachkundig“, zumal das Gros der höheren Verwaltungsbeamten des Reiches das Studium der Rechtswissenschaften absolviert hatte (Luttenberger 2013: 76, 291). Selbst in Bereichen also, in denen dies eigentlich fernlag, sollten (vorgeblich) sachkundige Laien beteiligt werden. Das ist ein Indikator dafür, wie sehr „Sachkunde“ mit allen Implikationen, die sich an diesen Begriff im Hinblick auf wissenschaftliche Objektivität und Unhinterfragbarkeit knüpften, zu einer tendenziell inflationär verwendeten Chiffre für ein an die NS-Ideologie anschlussfähiges „lebensgesetzliches Recht“ geworden war.

In vielen Fällen blieb es bei Plan- und Gedankenspielen. Dennoch scheint es lohnend, die These näher zu prüfen, die Reformvorstöße hätten in eine tiefgreifende Veränderung der Gerichtslandschaft insgesamt münden können, wäre das NS-Regime von längerer Dauer gewesen. Die Initiativen geben Aufschluss über die Rechtsvorstellungen der Nationalsozialisten und das Wesen jener „eigene[n] Version der Laiengerichtsbarkeit“ des „Dritten Reichs“, von der die Forschung annimmt, sie hätte sich ausbilden können, hätten die „Erfordernisse des Krieges“ es nicht verhindert (inkl. Zitaten: Casper & Zeisel 1979: 27).

Wäre es dazu gekommen, wäre wohl auch die neuartige Methodik der Gesetzeserläuterung zunehmend zur Regel geworden. Angesichts der Tatsache, dass die Bestrebungen hin zu einer Stärkung des sachkundigen Laienrichtertums bis in die zweite Hälfte der 1930er Jahre über unterschiedlichste Rechtsgebiete hinweg sehr ausgeprägt waren, hätte dies auch Disziplinen weit über die medizinisch-eugenischen Wissenschaften hinaus betroffen.

## Gebiete und Grenzen der Verschränkung von Wissenschaft und Recht

Der erst ansatzweise beantworteten Frage, wie sich die nationalsozialistischen Initiativen, die Textgattung „Gesetzeskommentar“ zu reformieren, in der Praxis auswirkten, ist nicht in erster Linie mit rechtshistorischen Konzepten beizukommen. Es sind weniger die bislang in den Fokus genommenen Neukommentierungen überkommener Gesetze im nationalsozialistischen Sinne, die als Quellengrundlage einer Analyse der angestrebten Reform taugen. Vielmehr muss man den Blick auf Kommentierungen nach 1933 erlassener und nach 1945 wieder außer Kraft gesetzter, genuin nationalsozialistischer Gesetze richten. Diese Gesetze aber wurden häufig unter maßgeblicher Beteiligung sachkundiger juristischer Laien angewendet – und erläutert. So kam es zu einer Verschränkung der Rechtswissenschaft mit anderen Wissenschaften und Wissensbereichen, die ihren Niederschlag im Genre des Gesetzeskommentars fand.

Diese Textgattung war prädestiniert, zum Medium der besagten Verschränkung zu werden, weil sie ursprünglich mit den nationalsozialistischen Rechtsvorstellungen inkompatibel, für die Rechtspraxis jedoch unverzichtbar war. Daher galt es, sie an die NS-Rechtslehre anschlussfähig zu machen, indem der Kommentar zum Mittler zwischen den Rechtswissenschaften und den verstärkt in die Rechtsfindung eingebundenen Fachwissenschaften gemacht wurde. An die Stelle, wo in der Ära des Rechtspositivismus Wortlautauslegungen und rechtstheoretische Überlegungen gestanden hatten, traten im Kommentar nationalsozialistischer Prägung vorgeblich objektive wissenschaftliche Befunde, aus denen sich vermeintlich zwingende Konsequenzen ableiten ließen.

Auf den Kommentar als Gegenstand angewendet bietet Mitchell Ashs für die Analyse des Verhältnisses von Wissenschaften und Politik im Nationalsozialismus entwickeltes Verflechtungskonzept daher auch einen Schlüssel zum Verständnis der Wechselbeziehung von Wissenschaften und Recht im „Dritten Reich“. Das am Beispiel des Gütt/Rüdin/Ruttke entwickelte Modell einer Verschränkung von Wissenschaften und Recht im Kommentar ist somit auch auf andere Gesetzeserläuterungen anwendbar.

Ob es zu einem Gesetz (neben Erläuterungen „herkömmlicher Machart“) Kommentierungen „neuen Typs“ gab, hing davon ab, ob an seiner Anwendung sachkundige Laien als Entscheidungsinstanzen beteiligt waren. Bemerkenswert ist, dass die nationalsozialistischen Machthaber über die sachkundigen Laien die ideologiekonformen Auslegungsschriften „neuen Typs“ mitunter zu *de facto* amtlichen, somit für die Laienrichter und indirekt dadurch auch für die Juristen verbindlichen Kommentaren deklarierten. Ironischerweise bekam so jene Textgattung, die einige Nationalsozialisten 1934 noch verbieten wollten, letztlich einen höheren Stellenwert. Deshalb scheint es sinnvoll, Rechtskommentare – auch aus anderen Rechtsgebieten als dem „Rassenrecht“ – systematischer als bisher zu Quellen und zum Gegenstand der NS-Forschung zu machen.

In einem ersten Schritt wären neben den systematischen Grenzen auf Ebene der Entscheidungsinstanzen auch die Grenzen der Kommentierung „neuen Typs“ auf politischer Ebene zu untersuchen, die hier nur angedeutet sind. Zumindest auf dem Gebiet des „Rassenrechts“ überließen die NS-Machthaber den Medizinern nicht das Feld, sondern suchten sie einzuhegen (Yurdakul 2017: 133). In diesem Kontext ist eine Kontroverse bemerkenswert, die Gütt 1937/38 mit dem Reichsärzteführer Gerhard Wagner (1888–1939) austrug. Wagner kritisierte, der von Gütt mitverantwortete GzVeN-Kommentar lege das Gesetz zu extensiv aus und habe auch zu unerwünschten Erbgesundheitsverfahren gegen NSDAP-Mitglieder geführt (Bock 1986: 375f.). Bislang wurde dieser Konflikt, in dessen Verlauf Gütt in die Defensive geriet, fast ausschließlich unter machtpolitischen Gesichtspunkten betrachtet (Schmuhl 1987: 165). Wie wirkte er sich aber angesichts der Tatsache, dass Gütt einer der aktivsten Herausgeber neuartiger Kommentare war, auf die Bestrebungen hin zu einer Reform des Kommentarwesens insgesamt aus?

In methodologischer Hinsicht legen die obigen Ausführungen nahe, dass sich das Konzept Ashs auch für die Analyse der Verhältnisse anderer gesellschaftlicher Teilsysteme als nur der Wissenschaft und Politik fruchtbar machen lässt. Auch die Beziehungen zwischen Wissenschaften und Rechtswesen im Nationalsozialismus lassen sich mit ihm greifbar machen, ebenso zwischen Wissenschaften untereinander. Zugleich verweisen die Befunde darauf, dass im Zuge der Anwendung des Ash’schen Modells die Frage, „*wo* genau die relevanten Verknüpfungen“ (Ash 2016: 542) zwischen den gesellschaftlichen Teilsystemen zu suchen sind, weiter gefasst werden sollte. Neben Netzwerken (Ash 2016: 542) wären auch jene Kommunikationsforen zu thematisieren, die sich als „Medien der Ressourcenmobilisierung“ bezeichnen ließen: Inwieweit waren etwa bestimmte Textsorten – wie der juristische Kommentar – prädestiniert, als Scharniere zwischen den gesellschaftlichen Teilsystemen zu fungieren? Lassen sich hier wiederkehrende Muster ausmachen? Handelte es sich regelmäßig um Medien, die in ihrer überkommenen Form problematisch für den Nationalsozialismus waren?

## Danksagung

Für Anregungen und kritische Lektüre danke ich Sophie Arndt, Franziska Querengäßer, Rüdiger Hachtmann und Friedrich Steinle.

## Footnotes

1 Kästle arbeitet die zitierten Autoren als drei Hauptakteure der Debatte heraus (Kästle 2013: 433).

2 Dageförde (1934: 45); Seydel (1936); Kästle (2013: 432f.); Kästle-Lamparter (2016: 77f.).

3 Neben den bereits zitierten Untersuchungen weniger ausführlich Henne (2006: 354).

4 Eine recht ausführliche, wenn auch nicht vollständige Aufstellung von Gerichten, die im Nationalsozialismus unter Beteiligung von Laien entschieden, gibt Kross (1991: 265–289).

5 Vgl. beispielhaft nur für die Erbgesundheitsgerichte die entsprechenden Kapitel bei Braß (2004); Daum & Deppe (1991); Hennig (2000); Hinz-Wessels (2004); Heitzer (2005); Marnau (2003). Für weitere bis 1995 erschiene Literatur vgl. die sorgfältige Bibliografie von Beck (1995: 122–132).

6 Einzuschränken ist, dass § 1 Abs. 1 Buchst. d EGG den Indikationenkatalog des GzVeN indirekt aufnahm, indem er Eheschließungen verbot, wenn ein Verlobter an einer Erbkrankheit im Sinne dieses Gesetzes litt.

7 Dass dieses klassische Rechtsdenken in der Entstehungsphase auch an Erbgesundheitsgerichten noch verbreitet war, zeigt eine Äußerung des Mediziners Paul Nitsche (1876–1948). In einem Schreiben an Rüdin monierte der Dresdner Erbgesundheitsrichter in Anspielung auf den Strafrechtsgrundsatz „in dubio pro reo“: „Die Gerichte können vielfach von dem Grundsatz ‚in dubio pro aegroto‘ [im Zweifel für den Kranken] nicht loskommen“ (zit. nach Schmuhl 2016: 229).

8 So ist etwa bei Brandis mit Blick auf Leiden, die gemäß § 1 Abs. 1 Buchst. c eheuntauglich machten, lediglich vage von „geistigen Störungen […], die zu s c h w e r e r P s y c h o p a t h i e oder zu v e r b r e c h e r i s c h e m , d i e G e m e i n s c h a f t g e f ä h r d e n d e m V e r h a l t e n g e f ü h r t h a b e n [Hervorhebungen im Original]“ (Brandis 1936: 118), die Rede.

9 Siehe nur für die *Dermatologische Wochenschrift* sowie die Zeitschrift *Der Öffentliche Gesundheitsdienst* in Auswahl und ohne Anspruch auf Vollständigkeit: Spiethoff (1938: 1506); Schwarz (1938/39: 202); von Neureiter (1938/39: 305); Klose (1940/41: 332); Maerz (1941/42: 135).

10 Eine Ausnahme hiervon war der Reichsausschuss für Ehegenehmigungen. Er prüfte Anträge auf die Genehmigung von „Mischehen“. Auch dieses Gremium bestand jedoch nicht nur aus „Rassewissenschaftlern“ (zumeist Mediziner, die sich mit der Rassentheorie beschäftigten); Juristen waren ebenfalls Mitglieder (namentliche Aufstellung bei Meyer 1999: 169f.).

11 Beispiele stellen etwa die Erörterungen der Begriffe „Träger deutschen Blutes“ und „dem deutschen Volk artverwandt“ dar (GLM: 202).

12 Eine Ausnahme stellt die Auslegung des § 6 Erste AusfVO z. Blutschutzgesetz dar, auf die hier nicht näher eingegangen werden kann – die Bestimmung unterlag als einzige der Kontrolle durch die Medizinalbeamten der Gesundheitsämter, weshalb ihre Kommentierung wiederum der des EGG glich.
